# Plasticity of blood feeding behavior of* Anopheles* mosquitoes in Ethiopia: a systematic review

**DOI:** 10.1186/s13071-024-06493-1

**Published:** 2024-09-28

**Authors:** Temesgen Ashine, Abena Kochora, Hailu Shibru, Alemayehu Bekele, Muluken Assefa, Bedasa Gidisa, Nigatu Negash, David Weetman, Tadesse Awoke Ayele, Endalamaw Gadisa, Fekadu Massebo

**Affiliations:** 1 Department of Biology, Arba Minch University, Arba Minch, Ethiopia; 2 Malaria and NTD Research Division, Armauer Hansen Research Institute, Addis Ababa, Ethiopia; 3 Department of Vector Biology, Liverpool School of Tropical Medicine, Pembroke Place, Liverpool, L35QA UK; 4 University of Gondar, Gondar, Ethiopia

**Keywords:** *Anopheles* mosquitoes, Blood meal source, Host preference, Blood-feeding behavior, Ethiopia

## Abstract

**Background:**

The efficacy of vector control tools depends on the behavior of the vector species. Many studies have sought to determine the feeding behavior of* Anopheles* mosquitoes in different settings of Ethiopia. We have performed a systematic review aimed to generate pooled evidence on the overall and species-specific blood meal sources of *Anopheles* mosquitoes in Ethiopia.

**Methods:**

A search for relevant articles was performed in two electronic databases (PubMed and Science Direct) and three search engines (Google Scholar, Research Gate and Google) between 11 March and 2 April 2024. Following the initial identification of articles, we used EndNote X8 software and removed duplicate articles and screened the remaining articles by careful reading of their titles and abstracts. The full text of articles that passed this screening phase was retrieved, read and evaluated against predetermined selection criteria. The final decision for inclusion in the systematic review was made after a methodological quality check using the JBI critical appraisal checklist. All relevant data were extracted from tables, figures and texts of the included articles using a premade template in Excel, and the data were analyzed using Stata version 14 software.

**Results:**

Of the 2431 studies identified, 27 met the inclusion criteria; all were published between 1997 and 2024. At 215 data points (frequency of tests of each *Anopheles* species by location and method of mosquito collections), 18,771 *Anopheles* mosquitoes belonging to 23 species or species complexes were tested for blood meal sources. The commonest sources of blood meals for *Anopheles* mosquitoes were bovine (36.0%, *n* = 6758) and human (29.4%, *n* = 5520). Among the tested anophelines, *Anopheles (An.) arabiensis* accounted for 67.9% (*n* = 12,741), followed by *An. pharoensis*, *An. demeilloni* and *An. stephensi* at 10.0%, 5.6% and 4.4%, respectively. Overall, there was no difference in the mean proportion of *An. arabiensis* detected with domestic animal blood (33.4%, 95% confidence interval [CI] 32.4–34.4%) and those detected with human blood (31.8%, 95% CI 30.9–32.8%). However, a greater proportion of the outdoor collected *An. arabiensis* were found to feed on bovines (47.9%, 95% CI 35.3–60.6) compared to humans (12.9%, 95% CI 0.8–24.9, *P* < 0.01). The foraging ratio (FR), which accounts for host availability, was greater for bovines (FR = 0.7) than for humans (FR = 0.2) for *An. arabiensis,* indicating preferential feeding on bovine hosts. This host preference was supported by the host preference index (human:bovine = 0.4). *Anopheles pharoensis* was detected with a slightly higher human blood index (53.5%, *n* = 1005) compared to bovine blood index (45.2%, *n* = 849). In contrast, *An. demeilloni*, *An. coustani* and *An. marshalli* were detected with a higher bovine blood index. Recently invaded urban malaria vector, *An. stephensi* was found with a higher ovine blood index.

**Conclusions:**

Bovine and human hosts are common sources of a blood meal for *Anopheles* mosquitoes. In terms of host availability, *An. arabiensis* showed preferential feeding on bovines/cattle. Targeting domestic animals, bovines and ovines with endectocides could supplement current vector control interventions.

**Study registration:**

The protocol of this study was registered on the International Prospective Register of Systematic Reviews, registration no. CRD42024515725.

**Graphical Abstract:**

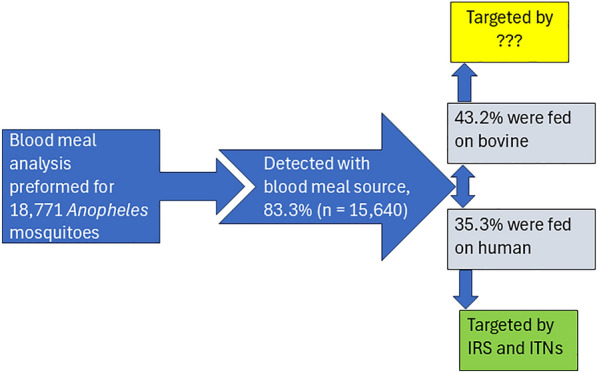

**Supplementary Information:**

The online version contains supplementary material available at 10.1186/s13071-024-06493-1.

## Background

*Plasmodium* parasites are transmitted to humans when an infected female *Anopheles* mosquito feeds on a susceptible host [1, 2]. Thus, in addition to supporting the sporogonic development of *Plasmodium* parasites, the host preference of *Anopheles* mosquitoes determines their vectorial capacity [3–5]. The major African malaria vectors mostly prefer feeding on human hosts to obtain blood for egg maturation [6]. In Ethiopia, multiple attempts have been made to determine the blood meal sources of malaria vectors [7–13]. Current evidence suggests that malaria vectors have a wide range of host preferences across different ecoepidemiological contexts.

To date, in Ethiopia, approximately 46 *Anopheles* mosquito species and subspecies have been recorded [14, 15]. However, only a few *Anopheles* species, including *An. arabiensis, An. pharoensis, An. funestus*, *An. nili* and *An. stephensi* have been implicated as vectors of malaria [16–19]. *Anopheles arabiensis* is the primary vector in most malaria-endemic areas in Ethiopia [16, 20], but the host preference (selective feeding behavior) of *An. arabiensis* has been found to vary across ecological gradients and epidemiological settings. *Anopheles arabiensis* has a human blood meal index (HBI) of 44% in the southwest region, 32.2% in the Southcentral region, 80% in irrigated villages and 73% in nonirrigated villages of central Ethiopia [7, 10, 19]. *Anopheles pharoensis* is of secondary importance in terms of being malaria vectors [16, 19], with *An. funestus, An. nili* and *An. stephensi* playing minor roles [8, 11, 17, 19].

Insecticide-treated nets (ITNs) and indoor residual spray (IRS) have been widely used to prevent malaria transmission by reducing human-vector contact inside human dwellings [21]. However, the effectiveness of IRS and ITNs is partly dependent on the biting and resting behavior of local malaria vectors. The greatest impacts of indoor-based vector control interventions can be achieved in areas where major malaria vectors feed and rest indoors [22, 23]. In addition, the peak biting time and location of local malaria vectors with nighttime human activities can affect the efficiency of these interventions [24].

In Ethiopia, several entomological studies have been conducted to determine the blood meal source and biting behavior of *Anopheles* mosquitoes. However, to our knowledge, no consolidated data on the overall and species-specific blood meal sources of *Anopheles* mosquitoes are currently available. A better understanding of the host feeding ranges and preferences of *Anopheles* mosquitoes can help to identify potential bottlenecks and gaps for future studies. This evidence could also support the tailoring of existing and new interventions to the behavior of local vectors for maximum effect. Therefore, the aim of this review is to summarize the overall and species-specific blood meal sources of *Anopheles* mosquitoes in Ethiopia.

## Methods

### Protocol registration

The protocol of this systematic review was registered in the International Prospective Register of Systematic Reviews with registration number CRD42024515725.

### Search strategies

A comprehensive and exhaustive literature search was performed by two authors to identify all relevant studies on blood meal sources of *Anopheles* mosquitoes in Ethiopia. The review question was: “What are the blood meal sources of *Anopheles* mosquito species in Ethiopia?”. Condition, context and population format were employed to develop the review question and search for relevant studies.

**Fig. 1:**
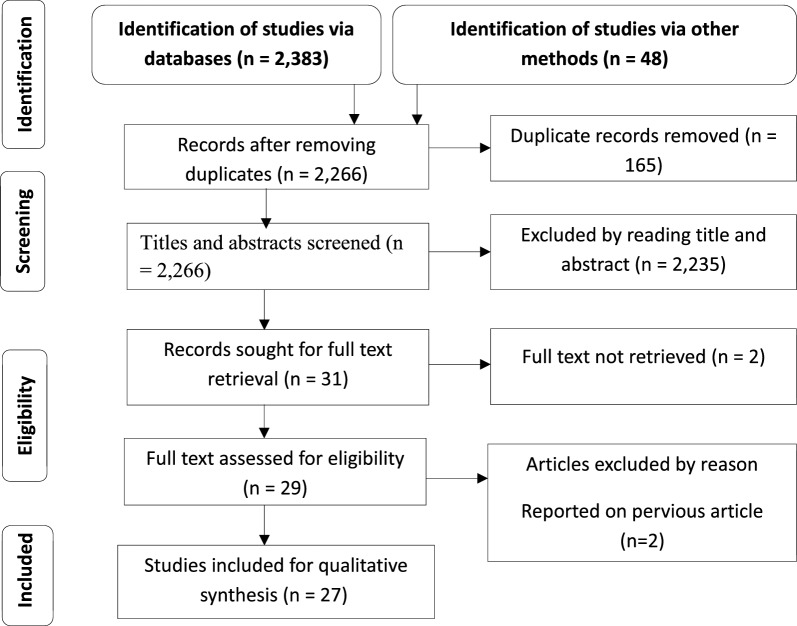
Preferred Reporting Items for Systematic Reviews and Meta-Analysis (PRISMA) flow diagram of the identification and selection of studies for inclusion in a systematic review of blood meal sources of* Anopheles* mosquitoes in Ethiopia

Between 15 March and 02 April 2024, published articles were retrieved from the following electronic databases: PubMed, Science Direct, the Google Scholar search engine and the Research Gate search engine. The reference lists of the identified studies were screened further to determine those studies eligible for inclusion in the systematic review. A separate Google search was performed for articles that fulfilled the selection criteria using predetermined search terms. This search was conducted using keywords and Medical Subject Headings (MeSH) with various combinations of search terms: ‘*Anopheles*,’ ‘malaria vector,’ ‘blood meal source,’ ‘blood meal index,’ ‘host preference,’ ‘blood meal origin’ and ‘Ethiopia.’ The search terms were used in combination with Boolean operators such as “OR” or “AND” (Additional file 1: Table S1). The Preferred Reporting Items for Systematic Reviews and Meta-Analysis (PRISMA) guidelines were followed (Fig. 1).

.

###  Eligibility criteria

Original articles that reported the blood meal sources of *Anopheles* mosquitoes from Ethiopia were considered for this review. Articles published in English reporting the source of the blood meal of *Anopheles* mosquitoes across all years of publication were considered. Studies in which the blood meal of *Anopheles* mosquitoes was tested for at least two host species using either direct enzyme-linked immunosorbent assay (ELISA) or multiplex-polymerase chain reaction (mPCR) were considered. The tested anopheline mosquito population needed to have been collected from the wild and freshly fed using standard entomological collection techniques. Findings on blood meal sources of non-*Anopheles* mosquitoes were excluded from the systematic review, and publications that were based on secondary data sources, including reviews and meeting proceedings, were not considered for inclusion.

### Study selection

Databases of all located studies was created on EndNote X8 (Bld 10,063; EndNote™; Clarivate, Philadelphia, PA, USA) and duplicate studies were removed. The initial assessment of the articles was performed by two reviewers independently by reading the titles and abstracts of each study. Full-text retrieval and further evaluation of relevant articles were performed by reading the full-text of each article. Studies without full texts were excluded after attempts to contact the primary author at least twice via email had no response. During the full-article review, reports that did not include adequate information on the blood meal source of *Anopheles* mosquitoes, reports of blood meal sources of unidentified anopheline species or articles that contained previously reported data were excluded. The level of agreement between two reviewers was evaluated using Kappa statistics and found to be 0.9, indicating the presence of good agreement between the reviewers. Any differences in article selection between assessors were settled by discussion and referral to a third author.

### Critical appraisal

The methodological quality and possibility of bias of the selected studies were assessed before inclusion in the review by performing a critical appraisal. Two authors independently evaluated the quality of each article using a standard quality assessment instrument adapted from the JBI critical appraisal checklist for studies reporting prevalence data [25]. Disagreements between two appraisers were settled by discussion and referral to a third author.

### Data extraction and management

Data extraction was independently conducted by two authors using a standardized data extraction tool that was adapted from the JBI data extraction tool for systematic reviews. Discrepancies in the data extracted by the two investigators were resolved by discussion and referral to a third reviewer. All required data, such as name of primary author, year of publication, year of data enumeration (mosquito collection), host density (proportion of specific hosts in the study area), study location (geographic location as reported in the original study), entomological sampling techniques used and location (indoor, outdoor or both) of mosquito collection, *Anopheles* mosquito species tested for blood meal source, number of mosquitoes tested and detected with specific host blood, host species tested for blood meal source detection, blood meal indices of specific *Anopheles* species for tested host species and methods used for detection of blood meal sources, were extracted into Excel format (Excel 2016; Microsoft Corp., Redmond, WA, USA).

The data extraction tool was developed so that the data were curated by data points. Data points are the frequency of test of specific *Anopheles* species in each of the reviewed articles by location (indoor, outdoor and both) and method of mosquito collection. Thus, the number of each *Anopheles* mosquito species tested and detected with specific host blood was extracted separately for each collection location and method (Table 1).

**Table 1:** Characteristics of the original studies (data points) included in the systematic review of blood meal origin/source of *Anopheles* mosquitoes, Ethiopia, 1997–2024

Characteristics of original studies	Categories	Data points by year of publication, *n* (%)			Total, *n* (%)^a^
		Before 2005	2005–2015	2016–2024	
Year of mosquito collection	Before 2005	14	3		17 (7.9)
	2005–2015		70	11	81 (37.7)
	2016–2024			117	117 (54.4)
	Total	14 (6.5)	73 (34.0)	128 (59.5)	215 (100)
Geographic range	Central	1	22	64	87 (40.5)
	Eastern		21	11	32 (14.9)
	Multiple locations		27		27 (12.6)
	Northern	1		25	26 (12.1)
	Northwestern	2		12	14 (6.5)
	Southcentral	7	3	2	12 (5.6)
	Southern			9	9 (4.2)
	Southwestern			5	5 (2.3)
	Western	3			3 (1.4)
Method of blood meal source detection	ELISA	14	73	110	197 (91.6)
	mPCR			18	18 (8.4)
Diversity and frequency of host species tested	Human & bovine	14	66	75	155 (72.1)
	Human, bovine, ovine & dog			5	5 (2.3)
	Human, bovine, ovine, dog & pig			43	43 (20.0)
	Human, bovine, ovine, dog & chicken			5	5 (2.3)
	Human, bovine, ovine, equine, dog & chicken		7		7 (3.3)
Location of mosquito collection	Indoor	10	40	38	88 (40.9)
	Outdoor	3	12	22	37 (17.2)
	Both	1	21	68	90 (41.9)
Mosquito collection techniques	CDC light trap	1	41	98	140 (45.0)
	Pyrethrum spray catches	18	17	29	64 (20.6)
	Prokopack aspirator			41	41 (13.2)
	Artificial pit shelter		12	16	28 (9.0)
	Mouth aspirator	13	3	5	21 (6.8)
	Clay Pot trap		1	9	10 (3.2)
	Backpack aspirator			2	2 (0.6)
	Animal bait tent trap			2	2 (0.6)
	Window exit trap			2	2 (0.6)
	Black resting box trap			1	1 (0.3)

*CDC* U.S. Center for Disease Control and Prevention, *ELISA* enzyme-linked immunosorbent assay, *mPCR* multiplex PCR

^a^*n* = frequency of data points

### Data synthesis

The data extracted using the Excel spreadsheet were exported to Stata software release 14 (StataCorp LP, College Station, TX, USA) for producing the statistical summaries. The characteristics of the original studies were summarized and presented. The test frequency of each *Anopheles* species by location and method of mosquito collection (data points) and species composition are presented graphically. The species-specific and overall indices of the blood meal of *Anopheles* mosquitoes were determined as the fraction of specific *Anopheles* species positive for a specific host blood to the number of that species tested for blood meal detection. A Kruskal–Wallis test was used to quantify the variation in the median proportion of *Anopheles* mosquitoes detected with human and bovine blood across collection locations (*An. arabiensis, An. coustani, An. demeilloni* and *An. pharoensis*) and year of mosquito collection (*An. arabiensis*). The foraging ratio (FR) was calculated as the percentage of each *Anopheles* species (*An. arabiensis, An. coustani, An. demeilloni, An. funestus* and *An. pharoensis*) detected with the blood of a particular host divided by the percentage of that particular host species in the total available host population [26, 27]; and was used to determine the avoidance (value < 1) or preference (value > 1) of a specific host species. Similarly, host preference indices (HPIs), an indicator of the observed proportion of fed mosquitoes on human hosts compared to bovine hosts divided by the expected comparative proportion of fed mosquitoes on these two hosts, were calculated to determine the preferential feeding of *Anopheles* mosquitoes on either human or bovine hosts [27, 28]. The mean proportion of human and bovine host blood was compared among *An. arabiensis* mosquitoes collected indoors and outdoors using the independent t-test.

## Results

### Literature search

The search for studies on the electronic databases and manually on search engines and other sources identified 2431 studies. After 165 duplicates had been removed, an initial screening of 2266 studies was conducted by reading the title and abstract of each study. This initial screening identified 31 studies that met the inclusion criteria; of these, full texts of 29 studies were successfully retrieved. An additional two studies were excluded following reading of the full texts (data had been presented in previous studies). Ultimately, 27 studies (with 215 data points/frequency of test of each species by method and locations of collection) that were published between 1997 and 2024 were included in this systematic review [8, 9, 11–13, 19, 29–49].

### Quality assessment

Overall, the 27 articles included in the systematic review were evaluated against the JBI appraisal criteria for quality and bias assessment. All critically appraised articles were judged to satisfy eight of the nine critical appraisal inquiries and met the predetermined cutoff for inclusion in the systematic review [20]. Since, the use of standard mosquito sampling method was considered to be one of the selection criteria, the critical appraisal inquiry that requests for appropriateness of sampling methods was not found to be applicable to studies selected for this systematic review ((Fig. 1; Additional file 1: Table S2)

### Characteristics of the original studies via data points

A total of 215 data points were identified in the 27 articles that included in this systematic review, of which 54.4% (*n* = 117) were based on entomological sampling after 2016. The data points were disproportionally distributed across geographical ranges/locations, with most (40.5%, *n* = 87) being from the central part of the country, followed by the eastern region (14.9%, *n* = 32). In earlier publications, based on mosquito collections up to 2005, the U.S. Centers for Disease Control and Prevention light trap (CDC LT) was used at one point for collecting host-seeking mosquitoes. Resting mosquito collections were made by a standard mouth aspirator at 13 data points and pyrethrum spray catches (PSCs) were used at 18 data points. The use of entomological sampling techniques that target either resting or host-seeking *Anopheles* mosquitoes has been more diversified in recent publications (mosquito collection since 2016). The frequency of using CDC LTs for collecting host-seeking mosquitoes has increased to 98 data points. Animal-baited tent traps have also been used for collecting host-seeking mosquitoes. The entomological techniques employed for collecting resting mosquitoes were expanded in more recent years to include prokopack and backpack aspirators, clay pots and black resting boxes. Overall, CDC LTs were the most frequent (45.0%, *n* = 140) entomological sampling technique, followed by PSCs (20.6%, *n* = 64) and prokopack aspirators (13.2%, *n* = 41). The latter two sampling methods were used for collecting resting mosquitoes indoors (PSCs) and both indoors and outdoors of human and animal shelters (prokopack aspirator) (Table 1).

Blood meal source determination among freshly fed *Anopheles* mosquitoes followed standard procedures [50, 51]. At most (91.6%, *n* = 197) of the data points, blood meal source detection was performed by ELISA. Two host species, human and bovine blood meals, were tested across all of the data points. One-fourth of the data points were tested for goat and dog blood meals; pigs, chickens and equines were tested less frequently.

### Abundance and composition of *Anopheles* species

**Fig. 2:**
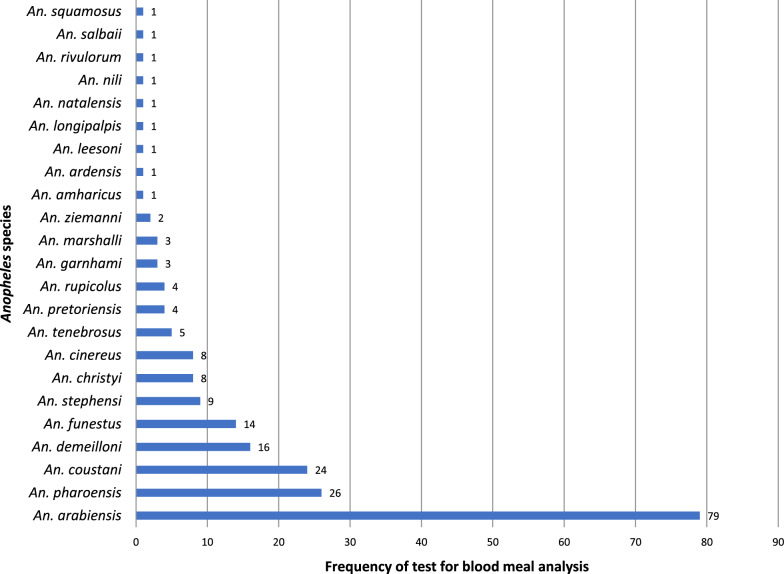
Frequency of occurrence of *Anopheles* mosquito species in blood meal sources analysis in the original studies included in the systematic review, Ethiopia, 1997–2024

A total of 18,771 freshly fed *Anopheles* mosquitoes, belonging to 23 species or species complexes, were tested for blood meal source detection (Table 2). *Anopheles arabiensis*, the primary vector of malaria in most malaria-endemic regions of Ethiopia, accounted for 67.9% (*n* = 12,741) of the tested *Anopheles* mosquito species for blood meal source detection, followed by *An. pharoensis*, 10.0% (*n* = 1879) and *An. demeilloni*, 5.6% (*n* = 1060). The new invasive urban malaria vector in the Horn of Africa, *An. stephensi*, accounted for 4.4% (*n* = 830) of the tested *Anopheles* mosquito species. A number of studies reported the tested mosquitoes as a species complex, including *Anopheles gambiae* sensu lato (*An. gambiae* s.l.), which accounted for 5.0% (*n* = 916) of the tested *Anopheles* mosquito species (Additional file 1: Table S3). However, molecular identification of 2271 morphologically identified *An. gambiae* s.l. confirmed that 95.6% (*n* = 2179) were *An. arabiensis* and 3.4% were not amplified; the remaining 14 mosquitoes were found to be *Anopheles amharicus* [30]. *Anopheles arabiensis* was the most frequently (*n* = 79) tested *Anopheles* mosquito species, followed by *An. pharoensis* (*n* = 26), *An. coustani* (*n* = 24)*, An. demeilloni* (*n* = 16), *An. funestus* (*n* = 14) and *An. stephensi* (*n* = 9) (Fig. 2).

**Table 2:** Origin of blood meal and blood meal indices (proportion of fed mosquitoes on specific host species) of *Anopheles* mosquitoes, Ethiopia, 1997–2024

*Anopheles* species	Human		Bovine		Ovine		Canine		MHBI, *n* (%)	UBI, *n* (%)	Animal, *n* (%)
	Tested, *n*	HBI, *n* (%)	Tested, *n*	BBI, *n* (%)	Tested, *n*	OBI, *n* (%)	Tested, *n*	CBI, *n* (%)			
*An. arabiensis*	12,741	4056 (31.8)	12,741	4107 (32.2)	2108	136 (6.5)	1884	15 (0.8)	1706 (13.4)	2450 (19.2)	4258 (33.4)
*An. pharoensis*	1879	1005 (53.5)	1879	849 (45.2)	55	2 (3.6)	55	0 (0.0)	44 (2.3)	185 (9.8)	851 (45.3)
*An. demeilloni*	1060	87 (8.2)	1060	687 (64.8)	22	2 (9.1)	22	0 (0.0)	63 (5.9)	201 (19.0)	689 (65.0)
*An. stephensi*	830	16 (1.9)	830	46 (5.5)	830	302 (36.4)	830	17 (2.0)	22 (2.7)	35 (4.2)	365 (44.0)
*An. coustani*	766	182 (23.8)	766	443 (57.8)	77	4 (5.2)	77	0 (0.0)	119 (15.5)	63 (8.2)	447 (58.4)
*An. marshalli*	656	68 (10.4)	656	308 (47.0)					175 (26.7)	105 (16.0)	308 (47.0)
*An. funestus*	337	27 (8.0)	337	40 (11.9)	14	0 (0.0)	14	0 (0.0)	201 (59.6)	37 (11.0)	40 (11.9)
*An. christyi*	189	46 (24.3)	189	109 (57.7)					18 (9.5)	16 (8.5)	109 (57.7)
*An. cinereus*	98	17 (17.3)	98	49 (50.0)					21 (21.4)	11 (11.2)	49 (50.0)
*An. squamosus*	54	0 (0.0)	54	12 (22.2)					39 (72.2)	3 (5.6)	12 (22.2)
*An. garnhami*	49	9 (18.4)	49	23 (46.9)					9 (18.4)	8 (16.3)	23 (46.9)
*An. pretoriensis*	35	0 (0.0)	35	33 (94.3)					0 (0.0)	2 (5.7)	33 (94.3)
*An. rupicolus*	22	0 (0.0)	22	22 (100)					0 (0.0)	0 (0.0)	22 (100)
*An. tenebrosus*	21	2 (9.5)	21	13 (61.9)	6	1 (16.7)	6	0 (0.0)	0 (0.0)	5 (23.8)	14 (66.7)
*An. amharicus*	14	2 (14.3)	14	3 (21.4)	14	1 (7.1)	14	0 (0.0)	0 (0.0)	8 (57.1)	4 (28.6)
*An. ziemanni*	6	2 (33.3)	6	3 (50.0)					1 (16.7)	0 (0.0)	3 (50.0)
*An. leesoni*	4	0 (0.0)	4	3 (75.0)					0 (0.0)	1 (25.0)	3 (75.0)
*An. nili*	3	1 (33.3)	3	2 (66.7)					0 (0.0)	0 (0.0)	2 (66.7)
*An. longipalpis*	2	0 (0.0)	2	2 (100)					0 (0.0)	0 (0.0)	2 (100)
*An. salbaii*	2	0 (0.0)	2	1 (50.0)					0 (0.0)	1 (50.0)	1 (50.0)
*An. ardensis*	1	0 (0.0)	1	1 (100)					0 (0.0)	0 (0.0)	1 (100)
*An. natalensis*	1	0 (0.0)	1	1 (100)					0 (0.0)	0 (0.0)	1 (100)
*An. rivulorum*	1	0 (0.0)	1	1 (100)					0 (0.0)	0 (0.0)	1 (100)
Total	18,771	5520 (29.4)	18,771	6758 (36.0)	3126	448 (14.3)	2902	32 (1.1)	2418 (12.9)	3131 (16.7)	7238 (38.6)

*Animal* Domestic animals (bovine, ovine and canine), *BBI* bovine blood meal index, *CBI* canine blood meal index, *HBI* human blood meal index, *MHBI* mixed human blood meal index, * n (%)* number of mosquitoes that tested positive (proportion of mosquitoes detected with specific host blood)* OBI* ovine blood meal index, *UBI* unknown blood meal proportion

### Common sources of blood meal for *Anopheles* species

Among the *Anopheles* mosquito specimens analyzed for blood meal source, host species were successfully identified in 83.3% (*n* = 15,640). Of the identified blood meal sources, bovine blood meal accounted for 36.0% (*n* = 6758), followed by human blood meal, accounting for 29.4% (*n* = 5520) (Table 2). A nonnegligible proportion of specimens (12.9%, *n* = 2418) were also identified with blood from two host species (mixed blood meal sources), especially bovines and humans. Blood from other host species was also detected in *Anopheles* mosquito specimens, including ovines (14.3%, *n* = 448) and canines (1.1%, *n* = 32). Among the reviewed studies, two reported the detection of chicken blood [36, 43], and one of these also reported equine host blood in a small proportion of tested *Anopheles* mosquitoes [43]. None of the tested mosquitoes tested positive for pig blood, and 16.7% (*n* = 3131) of the tested *Anopheles* mosquitoes had fed on an unidentified host species.

Blood from at least one of the tested *Anopheles* mosquito species was detected in each of the data points. Of the tested (*n* = 12,741) *An. arabiensis *mosquitoes, 31.8% (*n* = 4056) and 32.2% (*n* = 4107) had fed on human and bovine host blood, respectively; the remaining proportion of *An. arabiensis* had fed on unidentified (19.2%, *n* = 2450), mixed (13.4%, *n* = 1706), ovine and canine host blood. Overall, there was no difference in the mean proportion of *An. arabiensis* detected with domestic animal (bovine, ovine and canine) blood (33.4%, 95% confidence interval [CI] 32.4–34.4%) compared with human blood (31.8%, 95% CI 30.9–32.8%). A slightly higher proportion of *An. pharoensis,* a malaria vector of secondary importance in terms of malaria transmission in Ethiopia, was detected with human blood content (53.5%, *n* = 1005) compared to bovine blood content (45.2%, *n* = 849), with most of the mosquitoes collected inside human houses. However, a higher bovine blood meal index (BBI) compared to the HBI was recorded for *An. demeilloni* (64.8% vs 8.2%), *An. coustani* (57.8% vs 23.8%) and *An. marshalli* (47.0% vs 10.4%), bovine and human, respectively. The blood meal indices of the invasive urban malaria vector *An. stephensi* were higher for ovines (36.4%, *n* = 302) than for bovines (5.5%, *n* = 46) and humans (1.9%, *n* = 16). Most (59.9%, *n* = 202) of the tested *An. funestus* were detected with mixed blood meals (human and bovine) (Table 2).

The median proportions of HBIs and BBIs varied across collection locations for *An. arabiensis*. The HBI was higher among *An. arabiensis* mosquitoes collected indoors (median HBI 32.0, interquartile range [IQR] 14.1, 50.0) than among those collected outdoors (median 3.4, IQR 1.9, 18.5; *P* < 0.01). In contrast, the BBI was higher among *An. arabiensis* collected outdoors (median 48.4, IQR 42.8, 55.5) compared to those collected indoors (median 20.8, IQR 6.8, 44.1; *P* < 0.01) (Table 3). However, there was no difference in the blood meal index of *An. pharoensis* that fed on bovine hosts across collection locations, indoors (median 55.8, IQR 0.0, 70.0) and outdoors (median 83.3, IQR 83.3, 83.3, *P* = 0.31).

**Table 3:** Variations in human and bovine blood meal indices of the four most abundant *Anopheles* mosquito species across collection locations, Ethiopia, 1997–2024

*Anophele*s species	Collection location	*N*	Human blood meal index			Bovine blood meal index		
			*n* (%)	Median (IQR)	*P* value	*n* (%)	Median (IQR)	*P* value
*An. arabiensis*	Outdoors	2490	264 (10.6)	3.4 (1.9, 18.5)	< 0.01	1309 (52.6)	48.4 (42.8, 55.5)	< 0.01
	Indoors	5741	1450 (25.3)	32.0 (14.1, 50.0)		1596 (27.8)	20.8 (6.8, 44.1)	
*An. coustani*	Outdoors	64	0 (0.0)	0.0 (0.0, 0.0)	0.50	62 (96.9)	94.9 (91.7, 98.1)	0.18
	Indoors	47	2.0 (4.3)	0.0 (0.0, 33.3)		9 (19.1)	5.6 (0.0, 66.7)	
*An. demeilloni*	Outdoors	32	2 (6.3)	0.0 (0.0, 9.1)	0.49	23 (71.9)	77.8 (68.2, 100.0	0.34
	Indoors	738	75 (10.2)	2.9 (0.0, 9.8)		492 (66.7)	66.6 (5.0, 72.9)	
*An. pharoensis*	Outdoors	6	0 (0.0)	0.0 (0.0, 0.0)	0.19	5 (83.3)	83.3 (83.3, 83.3)	0.31
	Indoors	280	58 (20.7)	18.9 (10.0, 50.0)		151 (53.9)	55.8 (0.0, 70.0)	

*IQR* interquartile range, *N* number of mosquitoes tested, *n* number of mosquitoes detected with blood meal

The preferential feeding of *An. arabiensis* was determined by comparing the mean proportion of *An. arabiensis* that fed on bovine and human hosts. The mean proportion of *An. arabiensis* that fed on bovine hosts among outdoor catches (47.9, 95% CI 35.3–60.6) was significantly higher than the proportion that fed on human hosts (12.9, 95% CI 0.8–24.9) (*t*_(22)_ = − 4.4, *P* < 0.01) (Table 4). However, there was no statistically significant difference between the BBIs (26.3, 95% CI 18.8–33.9) and HBIs (34.7, 95% CI 26.7–42.7) (*t*_(68)_ = 1.5, *P* = 0.1) among *An. arabiensis* collected indoors.

**Table 4:** Preferred blood meal sources of *Anopheles arabiensis* across collection locations (t-test), Ethiopia, 1997–2024

Collection location	Blood meal source	Mean proportion (95% CI)	*t*-value	*df*	*P* value
Indoors	Human	34.7 (26.7, 42.7)	1.5	68.0	0.1
	Bovine	26.3 (18.8, 33.9)			
Outdoors	Human	12.9 (0.8, 24.9)	− 4.4	22.0	< 0.01
	Bovine	47.9 (35.3, 60.6)			
Indoors and outdoors	Human	21.8 (11.7, 32.0)	0.8	62.0	0.4
	Bovine	17.5 (12.6, 22.3)			
Overall	Human	26.2 (20.4, 32.0)	0.04	156	0.97
	Bovine	26.0 (21.3, 30.7)			

*CI* Confidence interval, *df Degree of freedom *

The human:bovine:ovine host ratios among the studies that surveyed the host population were 4.5:2.6:1.0, respectively. Thus, a comparison of the feeding preference of *An. arabiensis* was preformed among the most common host species (human and bovine) in the population and blood meal source tests. The FR of *An. arabiensis* for the bovine host (FR 0.7) was greater than that for the human host (FR 0.2). This preferential feeding of *An. arabiensis* on bovine hosts over humans was confirmed by the HPI (human:bovine 0.4) (Table 5).

**Table 5:** Foraging ratio and host preference index of five abundant *Anopheles* mosquito species, Ethiopia, 1997–2024

*Anopheles* species	Host	Mean host density	HBI	BBI	Foraging ratio		HPI (Human: Bovine
					Human	Bovine	
*An. arabiensis*	Human	54.7	13.6		0.2		0.4
	Bovine	45.4		31.0		0.7	
*An. coustani*	Human	54.0	1.2		0.0		0.1
	Bovine	46.0		7.3		0.2	
*An. demeilloni*	Human	59.1	0.0		0.0		0.0
	Bovine	40.9		4.5		0.1	
*An. funestus*	Human	64.4	0.0		0.0		0.0
	Bovine	35.6		6.4		0.2	
*An. pharoensis*	Human	61.5	12.0		0.2		0.2
	Bovine	38.5		48.0		1.2	

*BBI* Bovine blood meal index, *HBI* human blood meal index, *HPI* host preference index

Most (58.0%, *n* = 124) of the test results for blood meal source detection were reported by collection location, either indoors (40.9%, *n* = 88) or outdoors (17.2%, *n* = 37). However, 42.0% (*n* = 90) of the reported test results were not segregated by collection locations (Additional file 1: Table S3).

The overall proportion of *An. arabiensis* that fed on human hosts varied across the years of mosquito collection: before 2005 (median 26.3, IQR 9.3, 46.1), between 2005 and 20,115 (median 32.0, IQR 16.8, 66.7) and after 2015 (median 8.9, IQR = 0.0, 16.7; *P* = 0.01). However, this variation was not evident when the analysis was stratified according to the collection location. The proportion of *An. arabiensis* that fed on human hosts indoors did not vary across the years of mosquito collection: before 2005 (median 39.3, IQR 12.9, 48.1), 2005–2015 (median 29.8, IQR 13.2, 46.9) and after 2015 (median 50.0, IQR 14.3, 74.1; *P* = 0.53). Similarly, the was no difference in the HBI among *An. arabiensis* collected outdoors across the years of mosquito collection: before 2005 (median 2.6, IQR 0.9, 14.6), 2005–2015 (median 12.1, IQR 1.5, 43.9) and after 2015 (median 7.9, IQR 2.7, 14.2; *P* = 0.83) (Table 6). There was no variation in the overall BBI for *An. arabiensis* across the years of mosquito collection: before 2005 (median 40.6, IQR 10.5, 44.2), 2005–2015 (median 28.2, IQR 16.7, 39.1) and after 2015 (median 14.2, IQR 2.9, 43.1; *P* = 0.16).

**Table 6:** Blood meal sources of *Anopheles* *arabiensis* across years and locations of mosquito collection, Ethiopia, 1997–2024

Host species	Collection location	Year of collection, median (IQR)			*P* value
		Before 2005	2005 to 2015	After 2015	
Human	Outdoors	2.6 (0.9, 14.6)	12.1 (1.5, 43.9)	7.9 (2.7, 14.2)	0.83
	Indoors	39.3 (12.9, 48.1)	29.8 (13.2, 46.9)	50.0 (14.3, 74.1)	0.53
	Both	55.2 (55.2, 55.2)	71.6 (59.6, 79.9)	2.9 (0.0, 10.1)	< 0.01
	Overall	26.3 (9.3, 46.1)	32.0 (16.8, 66.7)	8.9 (0.0, 16.7)	< 0.01
Bovine	Outdoors	45.6 (42.8, 48.3)	43.0 (16.7, 55.5)	59.0 (48.7, 75.7)	0.18
	Indoors	29 (3.9, 44.2)	22.9 (9.5, 39.3)	14.2 (0.6, 57.1)	0.98
	Both	32.3 (32.3, 32.3)	28.2 (23.9, 29.2)	11.1 (1.5, 24.1)	0.02
	Overall	40.6 (10.5, 44.2)	28.2 (16.7, 39.1)	14.2 (2.9, 43.1)	0.16

*IQR* Interquartile range

## Discussion

The use of vector control interventions has played a significant role in reducing the malaria burden since the beginning of the twenty-first century [39, 40]. These vector control interventions primarily consist of targeting *Anopheles* mosquitoes that seek human hosts and rest indoors (within human houses). Thus, an understanding of the feeding behavior of local malaria vectors is critical for the effective selection and implementation of additional vector control interventions. *Anopheles arabiensis*, the primary malaria vector across most malaria-endemic regions of Ethiopia, has been found to show varying preferences for host species [13, 19, 42, 44], but most studies have shown its preference for nonhuman vertebrates, especially cattle [13, 33, 35, 36, 38, 42].

We conducted a systematic review of *Anopheles* mosquito blood meal sources in Ethiopia and showed that *An. arabiensis* exhibits anthropozoophilic feeding behavior. This study revealed that *An. arabiensis* can feed on multiple host species, the proportions of which vary between locations (indoor vs outdoor) where mosquitoes are collected. *Anopheles arabiensis* collected inside human dwellings had higher HBIs than those collected outdoors, while those collected outdoors were found with a high proportion of BBIs. Our finding is in line with a previous study which showed that the proportion of mosquitoes detected with different blood meal sources is primarily influenced by the location of collection (indoors vs outdoors) [6]. A recent study also demonstrated that *An. arabiensis* is amenable for zooprophylaxis [52], which is also in line with blood meal source analysis. However, we are cautious of its potential zoopotentiation effect, as easy access to blood meal sources prolongs the infectious life and reproductive potential of mosquitoes [53]. Perhaps such opportunistic feeding behavior of *An. arabiensis* might explain its success in thriving as the dominant vector, especially in rural settings.

Although the availability of a host is thought to determine the feeding behavior of *Anopheles* mosquitoes, it seems that *An. arabiensis* prefers to feed on bovine hosts. However, this result was not adjusted for the proportion of individuals protected by the physical barrier or the knockdown effect of the chemical on ITNs and the repellence effect of IRS. The potential diverting effect of insecticidal vector control interventions applied indoors toward unprotected animal hosts could also have contributed to the observed feeding preference on bovine hosts. Nevertheless, a significant proportion of *An. arabiensis* that were collected from inside human dwellings were detected with bovine blood. This finding corroborates the long-standing culture of sharing houses with cattle in many rural areas of Ethiopia [45, 49]. With this biting behavior on animal hosts, *An. arabiensis* might not be easily contained by existing indoor-based interventions. *Anopheles pharoensis*, one of the anopheline mosquitoes of secondary importance for malaria transmission in Ethiopia, was detected to have a high HBI. However, this might also be due to the location of mosquito collection, as observed for *An. arabiensis*, with most of the tested *An. pharoensis* having been captured from inside human dwellings. Other abundant *Anopheles* mosquitoes of secondary importance for malaria transmission in Ethiopia were found to be highly zoophagic, feeding on bovine (*An. demeilloni, An. coustani,* and *An. marshalli*) and ovine (*An. stephensi*) blood meal sources.

The recorded low proportion of *Anopheles* mosquitoes with human blood in comparison to those with blood of domestic animals (bovine, ovine and canine) might be indicative of the bottleneck in the feeding behavior of malaria vectors that can be targeted with supplemental interventions. Existing evidence shows that the application of the major insecticidal vector control interventions, IRS and ITNs, leads mosquitoes to change their resting and feeding habits to avoid the effects of insecticides (behavioral resistance) and to achieve easy access to blood meals [54–56]. However, our findings do not support this evidence, since neither the BBIs nor the HBIs significantly varied among mosquitoes collected from the same locations, especially for *An. arabiensis*. Thus, for anthropozoophilic mosquitoes, easy access to blood meal sources from unprotected animal hosts may support longevity and density and thus contribute to ongoing malaria transmission. Therefore, pushing mosquitoes away from human hosts and driving them to nearby domestic animals by existing and novel interventions and then targeting them with endectocides may be a future direction for supplemental interventions.

A significant proportion of the tested *Anopheles* mosquitoes were found to feed on unidentified blood meal sources, and this proportion varied across species. This unidentified proportion might imply a lack of primers or antibodies for common host species in the testing procedure. In all of the reviewed studies, cattle (bovine) and human primers and/or antibodies were commonly included for detecting blood meal sources in *Anopheles* mosquitoes. Other host species, including goats, dogs, pigs and chickens, have been tested infrequently. None of the reviewed studies assayed *Anopheles* mosquitoes for blood meal sources for some common (camel and sheep) domestic animals, and only one study tested them for equine (donkey, horse) hosts in Ethiopia. Thus, future studies need to consider unbiased and inclusive testing of *Anopheles* mosquitoes to determine the most common and preferred sources of blood meals across transmission settings. The impact of testing method and location of mosquito collection on sources of blood meal were not fully evaluated in this review since most of the data points in reviewed articles were unsegregated.

To our knowledge, this systematic review is the first of its kind to summarize the blood meal source of *Anopheles* mosquitoes in Ethiopia. The findings provide valuable evidence on the common sources of blood for *Anopheles* mosquitoes and the bottleneck in the feeding behavior that can be targeted by supplemental interventions. However, the findings might need to be interpreted with caution due to some limitations of the study. Although much effort was made to identify all relevant studies, some studies that satisfied the inclusion criteria may have remained unidentified. This systematic review is based on open access articles which were published in English. The curated data points across location, method and year of mosquito collections may not be sufficiently robust to determine the variation in host preferences. This might be more pronounced due to the lack of standard data reporting format for blood meal analysis across studies.

In conclusion, blood meal sources were successfully identified in most of the tested *Anopheles* mosquito specimens, but a nonnegligible proportion were found to fed on the blood of unidentified hosts. Domestic animals, particularly bovines and ovines, were reported to be the most common nonhuman sources of blood for *Anopheles* mosquitoes. Although the blood meal indices of *An. arabiensis* did not vary significantly between bovine and human host species, the foraging ratio and host preference index revealed the preferential feeding on bovine hosts. This evidence reveals the potential of targeting domestic animals, especially cattle and goats, with endectocide and/or calls for innovative topical agents that target *Anopheles* mosquitoes that seek blood from nonhuman animals as a supplemental vector control strategy.

## Supplementary Information

**Additional file 1: Table S1. **Databases and search engines with the respective search teams and records identified for the systematic review**. Table S2.** Methodological quality and bias assessment results of original studies for the systematic review of blood meal sources of *Anopheles* mosquitoes in Ethiopia, 1997-2024. **Table S3.** All eligible studies and corresponding data points retrieved for the systematic review of blood meal sources of *Anopheles *mosquitoes, Ethiopia, 1997–2024.

## Data Availability

No datasets were generated or analyzed during the current study.

## Abbreviations

BBI: Bovine blood meal index
CDC: U.S. Centers for Disease Control and Prevention
ELISA: Enzyme-linked immunosorbent assay
FR: Foraging ratio
HBI: Human blood meal index
HPIs: Host preference indices
IRS: Indoor residual spray
ITNs: Insecticide-treated nets
MeSH: Medical subject headings
mPCR: Multiplex PCR
OBI: Ovine blood meal index
