# Supplementary material for: Plasticity of blood feeding behavior of Anopheles mosquitoes in Ethiopia: a systematic review
Source: Parasit Vectors. 2024 Sep 28;17:408. doi: 10.1186/s13071-024-06493-1 (PMC11439269; doi:10.1186/s13071-024-06493-1)
Supplement: Supplementary file 1 — Additional file 1: Table S1. Databases and search engines with the respective search teams and records identified for the systematic review. Table S2. Methodological quality and bias assessment results of original studies for the systematic review of blood meal sources of Anopheles mosquitoes in Ethiopia, 1997-2024. Table S3. All eligible studies and corresponding data points retrieved for the systematic review of blood meal sources of Anopheles mosquitoes, Ethiopia, 1997–2024. [file 13071_2024_6493_MOESM1_ESM.docx]

**Additional file 1**

**Table 1:** Databases and search engines with the respective search teams and records identified for the systematic review.

| **Database/search engines** | **Search terms** | **Records identified** |
| --- | --- | --- |
| PubMed | ("anopheles"[MeSH Terms] OR "anopheles"[All Fields]) OR (("malaria"[MeSH Terms] OR "malaria"[All Fields]) AND ("genetic vectors"[MeSH Terms] OR ("genetic"[All Fields] AND "vectors"[All Fields]) OR "genetic vectors"[All Fields] OR "vector"[All Fields])) AND (("blood"[Subheading] OR "blood"[All Fields] OR "blood"[MeSH Terms]) AND ("meals"[MeSH Terms] OR "meals"[All Fields] OR "meal"[All Fields]) AND "source"[All Fields]) OR (("blood"[Subheading] OR "blood"[All Fields] OR "blood"[MeSH Terms]) AND ("meals"[MeSH Terms] OR "meals"[All Fields] OR "meal"[All Fields]) AND origin[All Fields]) OR (("blood"[Subheading] OR "blood"[All Fields] OR "blood"[MeSH Terms]) AND ("meals"[MeSH Terms] OR "meals"[All Fields] OR "meal"[All Fields]) AND ("abstracting and indexing"[MeSH Terms] OR ("abstracting"[All Fields] AND "indexing"[All Fields]) OR "abstracting and indexing"[All Fields] OR "index"[All Fields])) OR (("blood"[Subheading] OR "blood"[All Fields] OR "blood"[MeSH Terms]) AND ("meals"[MeSH Terms] OR "meals"[All Fields] OR "meal"[All Fields]) AND preference[All Fields]) AND ("ethiopia"[MeSH Terms] OR "ethiopia"[All Fields]) AND "open access"[filter] | 2001 |
| Science Direct | (“Anopheles species”) OR ("Anopheles mosquito") OR ("malaria vector") AND ("blood meal source") OR ("blood meal index") OR ("blood meal origin") AND (Ethiopia) = 748 | 382 |
| Google Scholar | Varies combination of Anopheles species, Anopheles mosquito, malaria vector, blood meal source, blood meal index, blood meal origin and Ethiopia | 21 |
| Research Gate | Varies combination of Anopheles species, Anopheles mosquito, malaria vector, blood meal source, blood meal index, blood meal origin and Ethiopia | 17 |
| Reference lists | Not applicable | 6 |
| Google | Varies combination of Anopheles species, Anopheles mosquito, malaria vector, blood meal source, blood meal index, blood meal origin and Ethiopia | 4 |
| Total records |  | 2431 |

**Table 2**: Methodological quality and bias assessment result of original studies for the systematic review of blood meal sources of *Anopheles* mosquitoes in Ethiopia, 1997-2024.

| **SN** | **Lead author (Year)** | **Q1** | **Q2** | **Q3** | **Q4** | **Q5** | **Q6** | **Q7** | **Q8** | **Q9** |
| --- | --- | --- | --- | --- | --- | --- | --- | --- | --- | --- |
| 1 | Hadis et al. (1997) | Yes | NA | Yes | Yes | Yes | Yes | Yes | Yes | Yes |
| 2 | Abossie et al. (2024) | Yes | NA | Yes | Yes | Yes | Yes | Yes | Yes | Yes |
| 3 | Adugna et al. (2021) | Yes | NA | Yes | Yes | Yes | Yes | Yes | Yes | Yes |
| 4 | Akirso et al. (2024) | Yes | NA | Yes | Yes | Yes | Yes | Yes | Yes | Yes |
| 5 | Animut et al. (2013) | Yes | NA | Yes | Yes | Yes | Yes | Yes | Yes | Yes |
| 6 | Ashine et al. (2024) | Yes | NA | Yes | Yes | Yes | Yes | Yes | Yes | Yes |
| 7 | Assa et al. (2023) | Yes | NA | Yes | Yes | Yes | Yes | Yes | Yes | Yes |
| 8 | Balkew et al. (2021) | Yes | NA | Yes | Yes | Yes | Yes | Yes | Yes | Yes |
| 9 | Bamou et al. (2021) | Yes | NA | Yes | Yes | Yes | Yes | Yes | Yes | Yes |
| 10 | Carter et al. (2021) | Yes | NA | Yes | Yes | Yes | Yes | Yes | Yes | Yes |
| 11 | Degefa et al. (2021) | Yes | NA | Yes | Yes | Yes | Yes | Yes | Yes | Yes |
| 12 | Eba et al. (2021) | Yes | NA | Yes | Yes | Yes | Yes | Yes | Yes | Yes |
| 13 | Eshetu et al. (2023) | Yes | NA | Yes | Yes | Yes | Yes | Yes | Yes | Yes |
| 14 | Gari et al. (2016) | Yes | NA | Yes | Yes | Yes | Yes | Yes | Yes | Yes |
| 15 | Getachew et al. (2019) | Yes | NA | Yes | Yes | Yes | Yes | Yes | Yes | Yes |
| 16 | Habtewold et al. (2001) | Yes | NA | Yes | Yes | Yes | Yes | Yes | Yes | Yes |
| 17 | Kibret et al. (2012) | Yes | NA | Yes | Yes | Yes | Yes | Yes | Yes | Yes |
| 18 | Kibret et al. (2014) | Yes | NA | Yes | Yes | Yes | Yes | Yes | Yes | Yes |
| 19 | Kindu et al. (2018) | Yes | NA | Yes | Yes | Yes | Yes | Yes | Yes | Yes |
| 20 | Lulu et al. (1998) | Yes | NA | Yes | Yes | Yes | Yes | Yes | Yes | Yes |
| 21 | Massebo et al. (2015) | Yes | NA | Yes | Yes | Yes | Yes | Yes | Yes | Yes |
| 22 | Tadesse et al. (2021) | Yes | NA | Yes | Yes | Yes | Yes | Yes | Yes | Yes |
| 23 | Tirados et al. (2006) | Yes | NA | Yes | Yes | Yes | Yes | Yes | Yes | Yes |
| 24 | Tomas et al. (2022) | Yes | NA | Yes | Yes | Yes | Yes | Yes | Yes | Yes |
| 25 | Yewhalaw et al. (2014) | Yes | NA | Yes | Yes | Yes | Yes | Yes | Yes | Yes |
| 26 | Yohannes et al. (2005) | Yes | NA | Yes | Yes | Yes | Yes | Yes | Yes | Yes |
| 27 | Zemene et al. (2021) | Yes | NA | Yes | Yes | Yes | Yes | Yes | Yes | Yes |

**Quality assessment items**

1Q) Was the sample frame appropriate to address the target population?

2Q) Were study participants sampled in an appropriate way?

3Q) Was the sample size adequate?

4Q) Were the study subjects and the setting described in detail?

5Q) Was the data analysis conducted with sufficient coverage of the identified sample?

6Q) Were valid methods used for the identification of the condition?

7Q) Was the condition measured in a standard, reliable way for all participants?

8Q) Was there appropriate statistical analysis?

9Q) Was the response rate adequate, and if not, was the low response rate managed appropriately?

**0**

**0**

**27**

**Overall appraisal: Include Exclude Seek further info**

**Table 2:** All eligible studies and corresponding data points retrieved for systematic review of blood meal source of *Anopheles* mosquitoes, Ethiopia, 1997-2024.

| **Lead author (Year)** | **HH** | **BH** | **Place** | **Method** | **Year_2** | **Species** | **N** | **HP** | **BP** | **OP** | **DP** | **MP** | **UH** |
| --- | --- | --- | --- | --- | --- | --- | --- | --- | --- | --- | --- | --- | --- |
| Akirso et al. (2024) |  |  | Indoor | CDC/PK | 2022 | *An. gambiae s.l.* | 180 | 90 | 1 |  |  | 0 | 89 |
| Akirso et al. (2024) |  |  | Indoor | CDC/PK | 2022 | *An. pharoensis* | 2 | 1 | 1 |  |  | 0 | 0 |
| Abossie et al. (2024) |  |  | Both | CDC/PSC | 2021 | *An. arabiensis* | 147 | 28 | 34 | 12 | 1 | 6 | 64 |
| Abossie et al. (2024) |  |  | Both | CDC/PSC | 2021 | *An. amharicus* | 14 | 2 | 3 | 1 | 0 | 0 | 8 |
| Abossie et al. (2024) |  |  | Both | CDC/PSC | 2021 | *An. coustani* | 5 | 0 | 2 | 1 | 0 | 0 | 1 |
| Abossie et al. (2024) |  |  | Both | CDC/PSC | 2021 | *An. pharoensis* | 2 | 0 | 2 | 0 | 0 | 0 | 0 |
| Eshetu et al. (2023) |  |  | Both | CP/APS/PSC | 2020 | *An. demeilloni* | 277 | 10 | 162 |  |  | 10 | 95 |
| Eshetu et al. (2023) |  |  | Both | CP/APS/PSC | 2020 | *An. funestus* | 23 | 3 | 10 |  |  | 0 | 10 |
| Eshetu et al. (2023) |  |  | Both | CP/APS/PSC | 2020 | *An. gambiae s.l.* | 42 | 3 | 14 |  |  | 2 | 23 |
| Eshetu et al. (2023) |  |  | Both | CP/APS/PSC | 2020 | *An. longipalpis* | 2 | 0 | 2 |  |  | 0 | 0 |
| Eshetu et al. (2023) |  |  | Both | CP/APS/PSC | 2020 | *An. salbaii* | 2 | 0 | 1 |  |  | 0 | 1 |
| Assa et al. (2023) |  |  | Indoor | CDC | 2020 | *An. demeilloni* | 109 | 10 | 70 |  |  | 21 | 8 |
| Assa et al. (2023) |  |  | Indoor | CDC | 2020 | *An. arabiensis* | 10 | 6 | 2 |  |  | 0 | 2 |
| Assa et al. (2023) |  |  | Indoor | CDC | 2020 | *An. leesoni* | 4 | 0 | 3 |  |  | 0 | 1 |
| Assa et al. (2023) |  |  | Indoor | CDC | 2020 | *Unidentified* | 1 | 0 | 0 |  |  | 1 | 0 |
| Assa et al. (2023) |  |  | Indoor | PSC | 2020 | *An. demeilloni* | 17 | 3 | 9 |  |  | 2 | 3 |
| Assa et al. (2023) |  |  | Indoor | PSC | 2020 | *An. arabiensis* | 4 | 3 | 0 |  |  | 0 | 1 |
| Ashine et al. (2024) |  |  | Both | CDC/PK | 2023 | *An. arabiensis* | 211 | 15 | 56 | 10 | 5 | 13 | 104 |
| Ashine et al. (2024) |  |  | Both | CDC/PK | 2023 | *An. pharoensis* | 22 | 2 | 4 | 1 | 0 | 2 | 12 |
| Ashine et al. (2024) |  |  | Both | CDC/PK | 2023 | *An. tenebrosus* | 6 | 1 | 3 | 1 | 0 | 0 | 1 |
| Ashine et al. (2024) |  |  | Both | CDC/PK | 2023 | *An. arabiensis* | 13 | 0 | 2 | 0 | 0 | 0 | 10 |
| Ashine et al. (2024) |  |  | Both | CDC/PK | 2023 | *An. arabiensis* | 1 | 0 | 0 | 0 | 0 | 1 | 0 |
| Ashine et al. (2024) |  |  | Both | CDC/PK | 2023 | *An. stephensi* | 7 | 0 | 0 | 3 | 0 | 0 | 3 |
| Ashine et al. (2024) |  |  | Both | CDC/PK | 2023 | *An. stephensi* | 52 | 1 | 25 | 4 | 0 | 8 | 14 |
| Ashine et al. (2024) |  |  | Both | CDC/PK | 2023 | *An. stephensi* | 22 | 2 | 6 | 6 | 0 | 0 | 8 |
| Ashine et al. (2024) |  |  | Both | CDC/PK | 2023 | *An. stephensi* | 3 | 1 | 0 | 1 | 0 | 1 | 0 |
| Ashine et al. (2024) |  |  | Both | CDC/PK | 2023 | *An. arabiensis* | 18 | 3 | 2 | 0 | 0 | 1 | 12 |
| Ashine et al. (2024) |  |  | Both | CDC/PK | 2023 | *An. arabiensis* | 29 | 9 | 1 | 0 | 3 | 6 | 10 |
| Ashine et al. (2024) |  |  | Both | CDC/PK | 2023 | *An. arabiensis* | 14 | 0 | 0 | 2 | 0 | 0 | 5 |
| Ashine et al. (2024) |  |  | Both | CDC/PK | 2023 | *An. funestus* | 1 | 0 | 0 | 0 | 0 | 0 | 0 |
| Ashine et al. (2024) |  |  | Both | CDC/PK | 2023 | *An. coustani* | 2 | 0 | 0 | 1 | 0 | 0 | 0 |
| Ashine et al. (2024) |  |  | Both | CDC/PK | 2023 | *An. arabiensis* | 5 | 0 | 0 | 0 | 0 | 1 | 4 |
| Ashine et al. (2024) |  |  | Both | CDC/PK | 2023 | *An. coustani* | 6 | 0 | 0 | 0 | 0 | 0 | 4 |
| Ashine et al. (2024) |  |  | Both | CDC/PK | 2023 | *An. arabiensis* | 9 | 0 | 2 | 3 | 0 | 0 | 1 |
| Ashine et al. (2024) |  |  | Both | CDC/PK | 2023 | *An. coustani* | 2 | 0 | 0 | 0 | 0 | 0 | 0 |
| Ashine et al. (2024) |  |  | Both | CDC/PK | 2023 | *An. coustani* | 1 | 0 | 0 | 0 | 0 | 0 | 0 |
| Ashine et al. (2024) |  |  | Both | CDC/PK | 2023 | *An. arabiensis* | 3 | 0 | 0 | 1 | 0 | 0 | 1 |
| Ashine et al. (2024) |  |  | Both | CDC/PK | 2023 | *An. arabiensis* | 14 | 0 | 2 | 3 | 0 | 2 | 4 |
| Ashine et al. (2024) |  |  | Both | CDC/PK | 2023 | *An. arabiensis* | 42 | 0 | 2 | 8 | 0 | 6 | 25 |
| Ashine et al. (2024) |  |  | Both | CDC/PK | 2023 | *An. pharoensis* | 10 | 0 | 0 | 1 | 0 | 0 | 8 |
| Ashine et al. (2024) |  |  | Both | CDC/PK | 2023 | *An. arabiensis* | 4 | 0 | 1 | 0 | 0 | 0 | 3 |
| Ashine et al. (2024) |  |  | Both | CDC/PK | 2023 | *An. funestus* | 4 | 1 | 0 | 0 | 0 | 0 | 3 |
| Ashine et al. (2024) |  |  | Both | CDC/PK | 2023 | *An. arabiensis* | 112 | 10 | 10 | 6 | 0 | 6 | 60 |
| Ashine et al. (2024) |  |  | Both | CDC/PK | 2023 | *An. pharoensis* | 1 | 0 | 1 | 0 | 0 | 0 | 0 |
| Ashine et al. (2024) |  |  | Both | CDC/PK | 2023 | *An. coustani* | 1 | 1 | 0 | 0 | 0 | 0 | 0 |
| Ashine et al. (2024) |  |  | Both | CDC/PK | 2023 | *An. arabiensis* | 77 | 7 | 7 | 5 | 0 | 22 | 20 |
| Ashine et al. (2024) |  |  | Both | CDC/PK | 2023 | *An. pharoensis* | 2 | 1 | 0 | 0 | 0 | 0 | 0 |
| Ashine et al. (2024) |  |  | Both | CDC/PK | 2023 | *An. coustani* | 13 | 1 | 0 | 1 | 0 | 5 | 4 |
| Ashine et al. (2024) |  |  | Both | CDC/PK | 2023 | *An. stephensi* | 7 | 1 | 0 | 0 | 0 | 2 | 1 |
| Ashine et al. (2024) |  |  | Both | CDC/PK | 2023 | *An. arabiensis* | 15 | 1 | 0 | 1 | 0 | 3 | 6 |
| Ashine et al. (2024) |  |  | Both | CDC/PK | 2023 | *An. funestus* | 5 | 0 | 0 | 0 | 0 | 0 | 3 |
| Ashine et al. (2024) |  |  | Both | CDC/PK | 2023 | *An. arabiensis* | 9 | 1 | 0 | 0 | 0 | 3 | 5 |
| Ashine et al. (2024) |  |  | Both | CDC/PK | 2023 | *An. arabiensis* | 8 | 0 | 1 | 0 | 0 | 0 | 0 |
| Ashine et al. (2024) |  |  | Both | CDC/PK | 2023 | *An. funestus* | 4 | 0 | 1 | 0 | 0 | 0 | 0 |
| Ashine et al. (2024) |  |  | Both | CDC/PK | 2023 | *An. arabiensis* | 9 | 1 | 1 | 0 | 0 | 4 | 2 |
| Ashine et al. (2024) |  |  | Both | CDC/PK | 2023 | *An. coustani* | 6 | 2 | 0 | 0 | 0 | 0 | 4 |
| Tomas et al. (2022) |  |  | Both | CP/APS | 2019 | *An. arabiensis* | 260 | 12 | 119 |  |  | 12 | 117 |
| Tomas et al. (2022) |  |  | Both | CP/APS | 2019 | *An. pharoensis* | 2 | 1 | 0 |  |  | 0 | 1 |
| Tomas et al. (2022) |  |  | Both | CP/APS | 2019 | *An. tenebrosus* | 1 | 0 | 0 |  |  | 0 | 1 |
| Tomas et al. (2022) |  |  | Both | CP/APS | 2019 | *An. demeilloni* | 1 | 0 | 0 |  |  | 0 | 1 |
| Zemene et al. (2021) |  |  | Both | CDC/PSC | 2018 | *An. coustani* | 127 | 7 | 110 |  |  | 7 | 3 |
| Zemene et al. (2021) |  |  | Both | CDC/PSC | 2018 | *An. gambiae s.l.* | 40 | 21 | 11 |  |  | 6 | 2 |
| Zemene et al. (2021) |  |  | Both | CDC | 2018 | *An. pharoensis* | 17 | 4 | 13 |  |  | 0 | 0 |
| Tadesse et al. (2021) |  |  | Both | PSC/MA | 2019 | *An. stephensi* | 72 | 9 | 7 | 23 | 5 | 11 |  |
| Eba et al. (2021) |  |  | Indoor | PSC | 2016 | *An. arabiensis* | 631 | 89 | 316 | 4 | 1 | 89 | 68 |
| Degefa et al. (2021) | 39 | 32.2 | Indoor | CDC | 2018 | *An. arabiensis* | 24 | 4 | 17 | 2 | 0 | 1 | 0 |
| Degefa et al. (2021) | 39 | 32.2 | Indoor | CDC | 2018 | *An. pharoensis* | 10 | 1 | 7 | 0 | 0 | 1 | 1 |
| Degefa et al. (2021) | 39 | 32.2 | Indoor | CDC | 2018 | *An. coustani* | 4 | 0 | 4 | 0 | 0 | 0 | 0 |
| Degefa et al. (2021) | 39 | 32.2 | Indoor | PSC | 2018 | *An. arabiensis* | 28 | 4 | 17 | 2 | 0 | 1 | 3 |
| Degefa et al. (2021) | 39 | 32.2 | Indoor | PSC | 2018 | *An. pharoensis* | 8 | 1 | 5 | 0 | 0 | 0 | 2 |
| Balkew et al. (2021) |  |  | Both | BB/MA/CBT | 2019 | *An. stephensi* | 394 | 1 | 4 | 117 | 8 |  |  |
| Balkew et al. (2021) |  |  | Both | MA/CBT | 2019 | *An. stephensi* | 237 | 0 | 1 | 126 | 3 |  |  |
| Adugna et al. (2021) | 37.7 | 40 | Both | CDC | 2016 | *An. arabiensis* | 208 | 0 | 6 |  |  | 191 | 11 |
| Adugna et al. (2021) | 37.7 | 40 | Both | CDC | 2016 | *An. funestus* | 213 | 0 | 9 |  |  | 194 | 9 |
| Adugna et al. (2021) | 37.7 | 40 | Both | CDC | 2016 | *An. coustani* | 122 | 2 | 5 |  |  | 106 | 9 |
| Adugna et al. (2021) | 37.7 | 40 | Both | CDC | 2016 | *An. squamosus* | 54 | 0 | 12 |  |  | 39 | 3 |
| Adugna et al. (2021) | 37.7 | 40 | Both | CDC | 2016 | *An. cinereus* | 12 | 0 | 0 |  |  | 10 | 2 |
| Adugna et al. (2021) | 37.7 | 40 | Indoor | PSC | 2016 | *An. arabiensis* | 1 | 0 | 0 |  |  | 0 | 1 |
| Adugna et al. (2021) | 37.7 | 40 | Indoor | PSC | 2016 | *An. funestus* | 4 | 0 | 0 |  |  | 4 | 0 |
| Adugna et al. (2021) | 37.7 | 40 | Indoor | PSC | 2016 | *An. coustani* | 1 | 0 | 0 |  |  | 1 | 0 |
| Adugna et al. (2021) | 37.7 | 40 | Indoor | PSC | 2016 | *An. cinereus* | 2 | 0 | 0 |  |  | 1 | 1 |
| Carter et al. (2021) |  |  | Indoor | CDC/PSC | 2018 | *An. stephensi* | 36 | 1 | 3 | 22 | 1 |  | 9 |
| Bamou et al. (2021) |  |  | Both | CDC/PSC/BP/WT |  | *An. arabiensis* | 327 | 4 | 141 |  |  | 23 | 159 |
| Bamou et al. (2021) |  |  | Both | CDC/PSC/BP/WT |  | *An. pharoensis* | 44 | 0 | 18 |  |  | 1 | 25 |
| Kindu et al. (2018) |  |  | Both | CDC/PSC/APS | 2012 | *An. gambiae s.l.* | 7 | 2 | 2 |  |  | 0 | 3 |
| Kindu et al. (2018) |  |  | Both | CDC/PSC/APS/CP | 2012 | *An. cinereus* | 10 | 1 | 9 |  |  | 0 | 0 |
| Kindu et al. (2018) |  |  | Both | CDC/APS | 2012 | *An. demeilloni* | 12 | 0 | 10 |  |  | 1 | 1 |
| Gari et al. (2016) |  |  | Indoor | CDC | 2013 | *An. arabiensis* | 24 | 15 | 6 |  |  | 3 | 0 |
| Gari et al. (2016) |  |  | Indoor | CDC | 2013 | *An. pharoensis* | 7 | 6 | 0 |  |  | 1 | 0 |
| Gari et al. (2016) |  |  | Indoor | CDC | 2013 | *An. ziemanni* | 3 | 2 | 1 |  |  | 0 | 0 |
| Gari et al. (2016) |  |  | Indoor | PSC | 2013 | *An. arabiensis* | 48 | 35 | 10 |  |  | 3 | 0 |
| Gari et al. (2016) |  |  | Indoor | PSC | 2013 | *An. pharoensis* | 2 | 2 | 0 |  |  | 0 | 0 |
| Gari et al. (2016) |  |  | Outdoor | APS | 2013 | *An. arabiensis* | 19 | 4 | 10 |  |  | 3 | 2 |
| Gari et al. (2016) |  |  | Outdoor | APS | 2013 | *An. ziemanni* | 3 | 0 | 2 |  |  | 1 | 0 |
| Gari et al. (2016) |  |  | Outdoor | APS | 2013 | *An. funestus* | 1 | 0 | 1 |  |  | 0 | 0 |
| Massebo et al. (2015) | 61.3 | 20.4 | Indoor | CDC | 2010 | *An. arabiensis* | 988 | 94 | 70 |  |  | 644 | 180 |
| Massebo et al. (2015) | 61.3 | 20.4 | Indoor | CDC | 2010 | *An. marshalli* | 164 | 45 | 6 |  |  | 103 | 10 |
| Massebo et al. (2015) | 61.3 | 20.4 | Indoor | CDC | 2010 | *An. garnhami* | 7 | 4 | 0 |  |  | 2 | 1 |
| Massebo et al. (2015) | 61.3 | 20.4 | Indoor | CDC | 2010 | *An. pharoensis* | 7 | 1 | 0 |  |  | 2 | 4 |
| Massebo et al. (2015) | 61.3 | 20.4 | Indoor | CDC | 2010 | *An. tenebrosus* | 4 | 1 | 1 |  |  | 0 | 2 |
| Massebo et al. (2015) | 61.3 | 20.4 | Indoor | CDC | 2010 | *An. funestus* | 1 | 0 | 1 |  |  | 0 | 0 |
| Massebo et al. (2015) | 61.3 | 20.4 | Indoor | PSC | 2010 | *An. arabiensis* | 352 | 59 | 154 |  |  | 74 | 65 |
| Massebo et al. (2015) | 61.3 | 20.4 | Indoor | PSC | 2010 | *An. marshalli* | 56 | 9 | 23 |  |  | 18 | 6 |
| Massebo et al. (2015) | 61.3 | 20.4 | Indoor | PSC | 2010 | *An. garnhami* | 7 | 3 | 2 |  |  | 2 | 0 |
| Massebo et al. (2015) | 61.3 | 20.4 | Indoor | PSC | 2010 | *An. funestus* | 1 | 0 | 0 |  |  | 0 | 1 |
| Massebo et al. (2015) | 61.3 | 20.4 | Outdoor | APS | 2010 | *An. arabiensis* | 894 | 27 | 521 |  |  | 89 | 257 |
| Massebo et al. (2015) | 61.3 | 20.4 | Outdoor | APS | 2010 | *An. marshalli* | 436 | 14 | 279 |  |  | 54 | 89 |
| Massebo et al. (2015) | 61.3 | 20.4 | Outdoor | APS | 2010 | *An. garnhami* | 35 | 2 | 21 |  |  | 5 | 7 |
| Massebo et al. (2015) | 61.3 | 20.4 | Outdoor | APS | 2010 | *An. funestus* | 14 | 0 | 5 |  |  | 3 | 6 |
| Massebo et al. (2015) | 61.3 | 20.4 | Outdoor | APS | 2010 | *An. tenebrosus* | 1 | 0 | 0 |  |  | 0 | 1 |
| Yewhalaw et al. (2014) | 34.1 | 23.6 | Indoor | MA | 2010 | *An. arabiensis* | 220 | 6 | 15 | 15 | 0 |  |  |
| Yewhalaw et al. (2014) | 34.1 | 23.6 | Indoor | MA | 2010 | *An. coustani* | 10 | 0 | 0 | 0 | 0 |  |  |
| Yewhalaw et al. (2014) | 34.1 | 23.6 | Indoor | MA | 2010 | *An. demeilloni* | 20 | 0 | 1 | 1 | 0 |  |  |
| Yewhalaw et al. (2014) | 34.1 | 23.6 | Indoor | CDC | 2010 | *An. arabiensis* | 235 | 5 | 28 | 28 | 4 |  |  |
| Yewhalaw et al. (2014) | 34.1 | 23.6 | Indoor | CDC | 2010 | *An. coustani* | 27 | 0 | 3 | 1 | 0 |  |  |
| Yewhalaw et al. (2014) | 34.1 | 23.6 | Indoor | CDC | 2010 | *An. demeilloni* | 2 | 0 | 0 | 1 | 0 |  |  |
| Yewhalaw et al. (2014) | 34.1 | 23.6 | Outdoor | APS | 2010 | *An. arabiensis* | 6 | 0 | 0 | 0 | 1 |  |  |
| Kibret et al. (2014) |  |  | Both | CDC | 2010 | *An. arabiensis* | 2101 | 1678 | 593 |  |  |  | 16 |
| Kibret et al. (2014) |  |  | Both | CDC | 2010 | *An. pharoensis* | 992 | 631 | 491 |  |  |  | 21 |
| Kibret et al. (2014) |  |  | Both | CDC | 2010 | *An. coustani* | 215 | 107 | 156 |  |  |  | 9 |
| Kibret et al. (2014) |  |  | Both | CDC | 2010 | *An. funestus* | 58 | 21 | 9 |  |  |  | 2 |
| Kibret et al. (2014) |  |  | Both | CDC | 2010 | *An. arabiensis* | 234 | 171 | 56 |  |  |  | 13 |
| Kibret et al. (2014) |  |  | Both | CDC | 2010 | *An. pharoensis* | 71 | 42 | 38 |  |  |  | 5 |
| Kibret et al. (2014) |  |  | Both | CDC | 2010 | *An. coustani* | 29 | 11 | 23 |  |  |  | 4 |
| Kibret et al. (2012) |  |  | Both | CDC | 2007 | *An. arabiensis* | ##### | 149 | 38 |  |  |  | 21 |
| Kibret et al. (2012) |  |  | Both | CDC | 2007 | *An. pharoensis* | 231 | 138 | 69 |  |  |  | 40 |
| Kibret et al. (2012) |  |  | Both | CDC | 2007 | *An. coustani* | 70 | 31 | 43 |  |  |  | 11 |
| Kibret et al. (2012) |  |  | Both | CDC | 2007 | *An. funestus* | 6 | 2 | 3 |  |  |  | 2 |
| Kibret et al. (2012) |  |  | Both | CDC | 2007 | *An. arabiensis* | 111 | 89 | 29 |  |  |  | 10 |
| Kibret et al. (2012) |  |  | Both | CDC | 2007 | *An. pharoensis* | 154 | 102 | 42 |  |  |  | 32 |
| Kibret et al. (2012) |  |  | Both | CDC | 2007 | *An. coustani* | 33 | 13 | 20 |  |  |  | 5 |
| Kibret et al. (2012) |  |  | Both | CDC | 2007 | *An. funestus* | 2 | 0 | 1 |  |  |  | 1 |
| Kibret et al. (2012) |  |  | Both | CDC | 2007 | *An. arabiensis* | 89 | 57 | 26 |  |  |  | 18 |
| Kibret et al. (2012) |  |  | Both | CDC | 2007 | *An. pharoensis* | 28 | 18 | 9 |  |  |  | 3 |
| Kibret et al. (2012) |  |  | Both | CDC | 2007 | *An. coustani* | 15 | 4 | 10 |  |  |  | 2 |
| Kibret et al. (2012) |  |  | Both | CDC | 2007 | *An. arabiensis* | 47 | 28 | 15 |  |  |  | 11 |
| Kibret et al. (2012) |  |  | Both | CDC | 2007 | *An. pharoensis* | 17 | 8 | 6 |  |  |  | 5 |
| Kibret et al. (2012) |  |  | Both | CDC | 2007 | *An. coustani* | 8 | 1 | 3 |  |  |  | 4 |
| Tirados et al. (2006) | 62.5 | 37.5 | Indoor | PSC | 2004 | *An. arabiensis* | 250 | 96 | 78 |  |  | 58 | 18 |
| Tirados et al. (2006) | 62.5 | 37.5 | Outdoor | APS | 2004 | *An. arabiensis* | 820 | 204 | 341 |  |  | 154 | 121 |
| Tirados et al. (2006) | 5.6 | 94.4 | Indoor | PSC | 2004 | *An. arabiensis* | 43 | 4 | 23 |  |  | 16 | 0 |
| Habtewold et al. (2001) |  |  | Both | MA | 1999 | *An. arabiensis* | 96 | 53 | 31 | 10 |  | 2 | 0 |
| Habtewold et al. (2001) |  |  | Indoor | MA | 1999 | *An. arabiensis* | 64 | 27 | 26 | 9 |  | 2 | 0 |
| Habtewold et al. (2001) |  |  | Indoor | MA | 1999 | *An. arabiensis* | 64 | 2 | 45 | 15 |  | 1 | 1 |
| Lulu et al. (1998) |  |  | Indoor | MA | 1995 | *An. arabiensis* | 24 | 12 | 0 |  |  | 0 | 12 |
| Lulu et al. (1998) |  |  | Indoor | MA | 1995 | *An. arabiensis* | 19 | 5 | 2 |  |  | 2 | 10 |
| Lulu et al. (1998) |  |  | Indoor | MA | 1995 | *An. arabiensis* | 138 | 69 | 6 |  |  | 0 | 63 |
| Lulu et al. (1998) |  |  | Indoor | MA | 1995 | *An. arabiensis* | 55 | 30 | 1 |  |  | 0 | 24 |
| Lulu et al. (1998) |  |  | Indoor | MA | 1995 | *An. arabiensis* | 163 | 22 | 72 |  |  | 8 | 61 |
| Lulu et al. (1998) |  |  | Outdoor | MA | 1995 | *An. arabiensis* | 50 | 0 | 22 |  |  | 1 | 27 |
| Lulu et al. (1998) |  |  | Outdoor | MA | 1995 | *An. arabiensis* | 89 | 3 | 44 |  |  | 0 | 42 |
| Animut et al. (2013) |  |  | Indoor | CDC | 2010 | *An. arabiensis* | 422 | 135 | 165 |  |  | 57 | 65 |
| Animut et al. (2013) |  |  | Indoor | CDC | 2010 | *An. pharoensis* | 206 | 39 | 115 |  |  | 30 | 24 |
| Animut et al. (2013) |  |  | Indoor | CDC | 2010 | *An. arabiensis* | 64 | 28 | 22 |  |  | 8 | 6 |
| Animut et al. (2013) |  |  | Indoor | CDC | 2010 | *An. pharoensis* | 14 | 3 | 9 |  |  | 2 | 0 |
| Animut et al. (2013) |  |  | Indoor | CDC | 2010 | *An. christyi* | 9 | 1 | 6 |  |  | 1 | 1 |
| Animut et al. (2013) |  |  | Indoor | CDC | 2010 | *An. cinereus* | 10 | 2 | 6 |  |  | 2 | 0 |
| Animut et al. (2013) |  |  | Indoor | CDC | 2010 | *An. demeilloni* | 41 | 4 | 29 |  |  | 1 | 7 |
| Animut et al. (2013) |  |  | Indoor | CDC | 2010 | *An. arabiensis* | 6 | 3 | 1 |  |  | 0 | 1 |
| Animut et al. (2013) |  |  | Indoor | CDC | 2010 | *An. christyi* | 125 | 33 | 69 |  |  | 10 | 13 |
| Animut et al. (2013) |  |  | Indoor | CDC | 2010 | *An. cinereus* | 49 | 10 | 25 |  |  | 7 | 7 |
| Animut et al. (2013) |  |  | Indoor | CDC | 2010 | *An. demeilloni* | 471 | 54 | 325 |  |  | 26 | 65 |
| Hadis et al. (1997) |  |  | Indoor | MA | 1995 | *An. arabiensis* | 258 | 119 | 9 |  |  | 2 | 128 |
| Hadis et al. (1997) |  |  | outdoor | MA | 1995 | *An. arabiensis* | 174 | 3 | 82 |  |  | 1 | 88 |
| Hadis et al. (1997) |  |  | Indoor | MA | 1995 | *An. arabiensis* | 179 | 22 | 79 |  |  | 8 | 70 |
| Yohannes et al. (2005) | 25.6 | 74.4 | Indoor | CDC/PSC | 2000 | *An. arabiensis* | 194 | 78 | 52 |  |  | 64 |  |
| Animut et al. (2013) |  |  | Indoor | PSC | 2010 | *An. arabiensis* | 723 | 228 | 285 |  |  | 88 | 122 |
| Animut et al. (2013) |  |  | Indoor | PSC | 2010 | *An. pharoensis* | 16 | 4 | 7 |  |  | 4 | 1 |
| Animut et al. (2013) |  |  | Indoor | PSC | 2010 | *An. arabiensis* | 114 | 32 | 56 |  |  | 8 | 18 |
| Animut et al. (2013) |  |  | Indoor | PSC | 2010 | *An. pharoensis* | 7 | 0 | 6 |  |  | 1 | 0 |
| Animut et al. (2013) |  |  | Indoor | PSC | 2010 | *An. christyi* | 1 | 0 | 1 |  |  | 0 | 0 |
| Animut et al. (2013) |  |  | Indoor | PSC | 2010 | *An. cinereus* | 2 | 1 | 1 |  |  | 0 | 0 |
| Animut et al. (2013) |  |  | Indoor | PSC | 2010 | *An. demeilloni* | 1 | 0 | 0 |  |  | 0 | 1 |
| Animut et al. (2013) |  |  | Indoor | PSC | 2010 | *An. arabiensis* | 4 | 1 | 0 |  |  | 1 | 2 |
| Animut et al. (2013) |  |  | Indoor | PSC | 2010 | *An. christyi* | 37 | 10 | 18 |  |  | 7 | 2 |
| Animut et al. (2013) |  |  | Indoor | PSC | 2010 | *An. cinereus* | 12 | 2 | 8 |  |  | 1 | 1 |
| Animut et al. (2013) |  |  | Indoor | PSC | 2010 | *An. demeilloni* | 70 | 4 | 51 |  |  | 1 | 14 |
| Animut et al. (2013) |  |  | Outdoor | APS | 2010 | *An. arabiensis* | 3 | 2 | 1 |  |  | 0 | 0 |
| Animut et al. (2013) |  |  | Outdoor | APS | 2010 | *An. christyi* | 1 | 1 | 0 |  |  | 0 | 0 |
| Animut et al. (2013) |  |  | Outdoor | APS | 2010 | *An. christyi* | 2 | 1 | 1 |  |  | 0 | 0 |
| Animut et al. (2013) |  |  | Outdoor | APS | 2010 | *An. cinereus* | 1 | 1 | 0 |  |  | 0 | 0 |
| Animut et al. (2013) |  |  | Outdoor | APS | 2010 | *An. demeilloni* | 22 | 2 | 15 |  |  | 0 | 5 |
| Getachew et al. (2019) |  |  | Indoor | CDC | 2016 | *An. gambiae s.l* | 113 | 66 | 16 |  |  | 5 | 26 |
| Getachew et al. (2019) |  |  | Indoor | CDC | 2016 | *An. pretoriensis* | 3 | 0 | 2 |  |  | 0 | 1 |
| Getachew et al. (2019) |  |  | Indoor | CDC | 2016 | *An. coustani* | 3 | 1 | 2 |  |  | 0 | 0 |
| Getachew et al. (2019) |  |  | Indoor | CDC | 2016 | *An. demeilloni* | 4 | 0 | 4 |  |  | 0 | 0 |
| Getachew et al. (2019) |  |  | Indoor | CDC | 2016 | *An. rupicolus* | 1 | 0 | 1 |  |  | 0 | 0 |
| Getachew et al. (2019) |  |  | Indoor | CDC | 2016 | *An. pharoensis* | 1 | 0 | 1 |  |  | 0 | 0 |
| Getachew et al. (2019) |  |  | Indoor | CDC | 2016 | *An. gambiae s.l* | 21 | 3 | 12 |  |  | 0 | 6 |
| Getachew et al. (2019) |  |  | Indoor | CDC | 2016 | *An. pretoriensis* | 1 | 0 | 1 |  |  | 0 | 0 |
| Getachew et al. (2019) |  |  | Indoor | CDC | 2016 | *An. coustani* | 2 | 1 | 0 |  |  | 0 | 1 |
| Getachew et al. (2019) |  |  | Indoor | CDC | 2016 | *An. demeilloni* | 3 | 0 | 3 |  |  | 0 | 0 |
| Getachew et al. (2019) |  |  | Indoor | CDC | 2016 | *An. rupicolus* | 1 | 0 | 1 |  |  | 0 | 0 |
| Getachew et al. (2019) |  |  | Indoor | CDC | 2016 | *An. rivulorum* | 1 | 0 | 1 |  |  | 0 | 0 |
| Getachew et al. (2019) |  |  | Outdoor | CDC | 2016 | *An. gambiae s.l* | 76 | 12 | 36 |  |  | 1 | 27 |
| Getachew et al. (2019) |  |  | Outdoor | CDC | 2016 | *An. pretoriensis* | 29 | 0 | 28 |  |  | 0 | 1 |
| Getachew et al. (2019) |  |  | Outdoor | CDC | 2016 | *An. coustani* | 52 | 0 | 51 |  |  | 0 | 1 |
| Getachew et al. (2019) |  |  | Outdoor | CDC | 2016 | *An. demeilloni* | 9 | 0 | 7 |  |  | 1 | 1 |
| Getachew et al. (2019) |  |  | Outdoor | CDC | 2016 | *An. christyi* | 13 | 0 | 13 |  |  | 0 | 0 |
| Getachew et al. (2019) |  |  | Outdoor | CDC | 2016 | *An. rupicolus* | 12 | 0 | 12 |  |  | 0 | 0 |
| Getachew et al. (2019) |  |  | Outdoor | CDC | 2016 | *An. pharoensis* | 6 | 0 | 5 |  |  | 0 | 1 |
| Getachew et al. (2019) |  |  | Outdoor | CDC | 2016 | *An. tenebrosus* | 9 | 0 | 9 |  |  | 0 | 0 |
| Getachew et al. (2019) |  |  | Outdoor | CDC | 2016 | *An. ardensis* | 1 | 0 | 1 |  |  | 0 | 0 |
| Getachew et al. (2019) |  |  | Outdoor | CDC | 2016 | *An. natalensis* | 1 | 0 | 1 |  |  | 0 | 0 |
| Getachew et al. (2019) |  |  | Outdoor | CDC | 2016 | *An. gambiae s.l* | 8 | 1 | 4 |  |  | 0 | 3 |
| Getachew et al. (2019) |  |  | Outdoor | CDC | 2016 | *An. pretoriensis* | 2 | 0 | 2 |  |  | 0 | 0 |
| Getachew et al. (2019) |  |  | Outdoor | CDC | 2016 | *An. coustani* | 12 | 0 | 11 |  |  | 0 | 1 |
| Getachew et al. (2019) |  |  | Outdoor | CDC | 2016 | *An. demeilloni* | 1 | 0 | 1 |  |  | 0 | 0 |
| Getachew et al. (2019) |  |  | Outdoor | CDC | 2016 | *An. christyi* | 1 | 0 | 1 |  |  | 0 | 0 |
| Getachew et al. (2019) |  |  | Outdoor | CDC | 2016 | *An. rupicolus* | 8 | 0 | 8 |  |  | 0 | 0 |
| Getachew et al. (2019) |  |  | Outdoor | CDC | 2016 | *An. nili* | 3 | 1 | 2 |  |  | 0 | 0 |
| Getachew et al. (2019) |  |  | Indoor | PSC | 2016 | *An. gambiae s.l.* | 20 | 15 | 2 |  |  | 1 | 2 |
| Getachew et al. (2019) |  |  | Outdoor | APS | 2016 | *An. gambiae s.l.* | 60 | 2 | 50 |  |  | 1 | 7 |
| Getachew et al. (2019) |  |  | Indoor | MA | 2016 | *An. gambiae s.l.* | 58 | 43 | 8 |  |  | 0 | 7 |
| Getachew et al. (2019) |  |  | Shelter | MA | 2016 | *An. gambiae s.l.* | 291 | 6 | 198 |  |  | 2 | 85 |

*Key) Year, year of publication; HH, proportion of human host; BH, proportion of bovine host; Place, Place of mosquito collection, Method, entomological techniques employed for mosquito collection; Year_2, year of mosquito collection, Species, Anopheles mosquito species tested for blood meal source; N, number of mosquito tested; HP, number of mosquitoes positive for human blood; BP, number of mosquitoes positive for bovine blood; OP, number of mosquitoes positive for ovine blood; DP, number mosquitoes positive for dog blood, MP, number mosquitoes positive for human mixed blood; and UH, number of mosquitoes fed on unknow host blood.*
